# Correspondence

**Published:** 1898-09

**Authors:** 


### CORRESPONDENCE.

ATLANTA, GA., August 5, 1898.

*Editor Atlanta Medical and Surgical Journal:*

I beg to state through your journal that in the announcement of the Atlanta College of Physicians and Surgeons for the session of 1898–9 the publishing of my name as "Professor of Physiology," in the Dental Department of that college, is not only totally unwarranted, but has been done in the face of the fact that I have positively declined said position. I have had no connection, immediate or remote, with the Atlanta College of Physicians and Surgeons. Very respectfully,

JOHN C. OLMSTED, M.D.

---

JAKIN, GA., July 20, 1898.

*Editor Atlanta Medical and Surgical Journal:*

I report this case because I have never seen one like it, nor have I ever read of one. On July 15 Mrs. B. gave birth to twins without any accident, the second child being born about thirty minutes after the first. Twelve hours later this child began to have fever, and twenty-four hours after birth, when I saw him, he had a temperature of 104.5. I could find no cause for the fever whatever. I saw the child again forty-eight hours after birth, and at that time he had but little fever, respiration very shallow and frequent, pulse could not be counted, tips of fingers and toes were blue, and also a bluish discoloration around mouth and eyes. The face was drawn and indicative of much suffering. Was said to have had several convulsions. I told the parents that I did not think the child could possibly live through the night. However, when I returned the next day he was much better, and has continued to improve, and is now well. The nurse told that the child continued to grow worse during the night, and at one time she thought it dead. Scarcely any medicine was given at all. What could have caused the fever so soon after birth?

Yours truly, H. G. MINTER.

---

BIRMINGHAM, ALA., August 9, 1898.

*Editor Atlanta Medical and Surgical Journal:*

I notice in your valuable journal of August issue, page 384, under head of Correspondence, that inquiry is made with reference to an editorial which appeared in January number of *Pediatrics.* I inclose a reply which was published in the *Alabama Medical and Surgical Age,* in the June issue, and I believe it is the only reply or comment on this editorial which has been published, at least it is the only one which has come under my observation.

I send it to you in accordance with your request, that you would like to publish comments on the subject.

I am, yours very truly, JOHN C. LEGRAND, M.D.

The following, by Dr. T. L. Robertson, of Birmingham, Ala.:

"After you assume the most universal ignorance of the laity on the subject, and largely the medical profession, you proceed to treat the subject as a matter of no great moment, except as it relates to the importance of accuracy of knowledge in these days, and in that interest alone I ask to intrude these statement. A knowledge of just where Dr. Farabery got the information upon which to base the statement that 'The Negro baby at the time of its birth is exactly the same color as its white brother' would prove a revelation to be much appreciated by many members of the medical profession who have spent a life of active service among the Negro race in the Southern States, without even a suspicion that they would be confronted with a white baby at birth by Negro parents. That colony of Soudanese Negroes, which afforded that eminent French physician an opportunity to settle the vexed question, must have been a peculiar lot, or the doctor was laboring under some mental or optical delusions to arrive at his deductions, since our ample experience with the new-born Negro baby justifies no such deductions. I have been actively in the practice of medicine, including obstetrics, in Alabama, without intermission, since the spring of 1858. Many others here have had an equally long and ample experience, to assume the attitude of authority on the subject under discussion, and our deductions would be that babies born of negro parents are black—some of them very black, others not quite so black as they get to be later on. Those born of mulattoes, or mixed bloods, vary according to the predominance of the white or black race; but however light the general surface may be, if negro blood exists, the scrotum in the male is usually black; the female genitals do not show such distinguishing features so clearly. The offspring from a light mulatto or white mother by black father will usually be very dark, and *vice versa.* I must confess it was a matter of great astonishment to me to find so many statements so far at variance with the facts in so short an article, when facilities for correct knowledge on the subject are at your door. You can learn very near home that it takes neither sunlight nor climate to make the negro baby black. He comes into the world black, and remains so, unless by disease the pigment is destroyed, which sometimes happens."

---

LOUISVILLE, July 10, 1898.

*Editor Atlanta Medical and Surgical Journal:*

It will be appreciated if you will insert the following notice in an early issue. Very truly yours,

HENRY E. TULEY, Secretary.

The twenty-fourth annual meeting of the Mississippi Valley Medical Association will be held at Nashville, Tenn., October 11–14, under the presidency of Dr. John Young Brown, of St. Louis, Mo.

This Association is second in size only to the American Medical Association, and has done most excellent scientific work in the past. The annual addresses will be made by Dr. James T. Whittaker, of Cincinnati, on Medicine, and by Dr. Geo. Ben Johnston, of Richmond, Va., on Surgery. The mere mention of the names of these gentlemen establishes the fact that the Association will hear two scholarly and scientific addresses.

Nashville is a most excellent convention city, and is well equipped with hotels; and, with the record of the meeting in Louisville in

1897 as an example, the local profession, under the leadership of Dr. Duncan Eve as Chairman of the Committee of Arrangements, has prepared to have a better meeting.

Already titles of papers are being received. These should be sent to the Secretary, Dr. Henry E. Tuley, No. 111 West Kentucky street, Louisville, Ky., as early as possibly to insure a good place on the program. Reduced rates on railroads will be granted on the certificate plan.

---

We have received the following letter from the permanent Secretary of American Medical Association, with the request that we publish the same. For fear that some of our readers may not remember the "standard of requirement" of the American Medical College Association, we publish this as taken from the Constitution of this Association, to which this communication refers:

PHILADELPHIA, June 30, 1898.

DEAR SIR—At the recent meeting of this Association the following was unanimously adopted:

WHEREAS, The American Medical Association did, at Detroit in 1892, unanimously resolve to demand of all the medical colleges of the United States the adoption and observance of a standard of requirements of all candidates for the degree of doctor of medicine which should in no manner fall below the minimum standard of the Association of American Medical Colleges; and

WHEREAS, This demand was sent officially by the Permanent Secretary to the deans of every medical college in the United States and to every medical journal in the United States, now therefore the American Medical Association gives notice that hereafter no professor or other teacher in, nor any graduate of any medical college in the United States, which shall after January 1, 1899, confer the degree of doctor of medicine or receive such degree on any conditions below the published standard of the Association of American Medical Colleges, be allowed to register as either delegate or permanent member of this Association.

*Resolved*, That the Permanent Secretary shall, within thirty days after this meeting, send a certified copy of these resolutions to the dean of each medical college in the United States and to each medical journal in the United States.

Respectfully yours,

WM. B. ATKINSON, Permanent Secretary.

### AMERICAN MEDICAL ASSOCIATION OF COLLEGES.

### ARTICLE III. OF THE CONSTITUTION.

Section 1. Members of this Association shall require of all matriculants an English composition of not less than 200 words in the handwriting of the applicant, and an examination by a committee of the Faculty or other lawfully constituted board of examiners, in higher arithmetic, algebra, elementary physics and Latin prose.

Sec. 2. Graduates or matriculates of reputable colleges or high schools of the first grade, or normal schools established by State authority, or those who may have successfully passed the entrance examination provided by the statutes of the State of New York, shall be exempt from the requirements of section 1.

Sec. 3. Students conditioned in one or more of the branches enumerated as requirements for matriculation, shall have time until the beginning of the second year to make up such deficiencies, provided, however, that students who fail in any of the branches required in this second examination shall not be admitted to a second course.

Sec. 4. Colleges granting final examination on elementary subjects to junior students, shall not issue certificates of such final examination, nor shall any member of this Association confer the degree of Doctor in Medicine upon any person who has not been first examined upon all the branches of the curriculum by the faculty of the college granting the degree.

Sec. 5. Candidates for the degree of Doctor of Medicine shall have attended three courses of graded instruction of not less than three separate years.

Sec. 6. Students who have matriculated in any regular college prior to July 1, 1892, shall be exempted from these requirements.

At the meeting held in Detroit the American Medical Association adopted the following:

*Resolved,* That this Association most heartily indorses the efforts of the Association of American Medical Colleges to advance the cause of medical education, and demands of the medical colleges of the United States the adoption and observance of a standard of requirements which shall not in any respect fall below the minimum standard requirements adopted by such college association.

---

### YELLOW FEVER "IMMUNES."

Dr. J. C. LeHardy, Health Officer at Savannah, Ga., who has practiced through seven epidemics of yellow fever, is of the opinion that there is no such person as a "yellow fever immune"; that any person, although he has had yellow fever, is still liable to contract the disease if exposed to it. Said Dr. LeHardy on the subject: "The general belief is that you cannot have yellow fever twice. This is an error, due to the want of actual knowledge of the disease. Some persons never have it, but I have treated others four times during one epidemic (three times with black vomit). I have treated others three times, and many twice. Again, I have treated, in 1854, persons who had the disease in 1820 and 1830. In 1848 I attended numbers who suffered again in 1854. In 1876, the last time we had the fever in Savannah, I had patients who had been victims of three previous epidemics.—*Med. Fortnightly.*

---

### STONEWALL JACKSON'S MEDICAL DIRECTOR AGAIN IN SERVICE.

Dr. Hunter McGuire, the famous surgeon of Richmond, Va., who was during the civil war medical director of Stonewall Jackson's corps, has accepted a position on the staff of Major-General Fitzhugh Lee and will accompany that general to the front, or wherever else the latter may be ordered, as soon as he recovers from a temporary illness with which he is now suffering. Dr. McGuire is at present sojourning with his family at his White Sulphur Springs cottage, the same which was at one time occupied by Mrs. U. S. Grant.—*North American Med. Review.*

